# Zebrafish as a translational filter for natural sleep modulators

**DOI:** 10.3389/fnut.2026.1913649

**Published:** 2026-08-17

**Authors:** Xiaohui Tan, Zemei Nong, Xiaohong Deng, Chenxin Xie, Ji Zhang, Liping Wang, Ganlin Chen

**Affiliations:** 1 Guangxi Subtropical Crops Research Institute, Guangxi Academy of Agricultural Sciences, Nanning, China; 2 Guangxi Key Laboratory of Quality and Safety Control for Subtropical Fruits, Guangxi Subtropical Crops Research Institute, Nanning, China; 3 Key Laboratory of Quality and Safety Control for Subtropical Fruit and Vegetable, Ministry of Agriculture and Rural Affairs, Nanning, China

**Keywords:** functional foods, natural products, neuropharmacology, nutritional intervention, sleep, translational filter, zebrafish

## Abstract

The escalating global burden of sleep disorders and their tight association with cognitive decline, metabolic abnormalities, and psychiatric comorbidities constitute a major public health challenge. Long term use of current chemically synthesized sedative hypnotics is constrained by dependence, tolerance, and cognitive side effects, creating an urgent demand for safer, mechanism traceable nutritional intervention strategies. Plant derived natural products, owing to their structural diversity, broad spectrum target potential, and favorable safety profiles, represent a critical reservoir of next generation sleep modulating functional factors for precision nutrition. However, their development has long been hindered by a systemic translational gap between high throughput *in vitro* screening and physiologically relevant *in vivo* validation, leaving numerous potentially bioactive molecules stranded at the preclinical stage. The zebrafish, as a genetically tractable vertebrate model, exhibits deep evolutionary conservation with mammals in sleep regulatory behavioral outputs, core neural circuits (monoaminergic, GABAergic, orexin/hypocretin, and melatonin systems), and molecular clock mechanisms, alongside unique technical advantages including optical transparency, rapid development, and cost effective scalability. Here, we narratively review the application of zebrafish in evaluating the sleep modulating activity of natural products, and propose a conceptual, forward-looking, integrated, scalable *in vivo* platform that unifies behavioral phenotyping, neural circuit interrogation, and molecular target validation into a coherent pipeline—from high throughput phenotypic hit identification to mechanism guided lead prioritization. By interfacing with artificial intelligence assisted behavioral analytics, human organoid validation, and cross species data integration, this framework bridges the structural discovery validation gap that has historically impeded natural product sleep research. Importantly, we position the zebrafish not as a direct surrogate for clinical efficacy, but as a proposed translational filter and hypothesis generating engine that may accelerate the conversion of plant derived, mechanism defined sleep promoters into reproducible, quantifiable paradigms for functional food development and precision nutritional intervention.

## 1 Introduction

Sleep disorders have become a global public health crisis. Their prevalence continues to rise in modern societies and is significantly associated with cognitive decline, metabolic abnormalities, and the risk of psychiatric diseases (1). Although, current chemically synthesized sedative hypnotics are effective for short term intervention, their long term use is prone to dependence, tolerance, and cognitive side effects, which severely limit their sustainable application (2, 3). Consequently, there is an urgent need to explore novel, safe, sustainable, and mechanistically traceable sleep intervention strategies.

Plant derived natural products, owing to their diverse chemical structures and broad spectrum target potential, are regarded as a key source of next generation sleep regulating molecules. However, their development has long been constrained by a systemic bottleneck, most existing studies rely on *in vitro* screening or small scale animal models, making it difficult to achieve high throughput discovery and physiologically relevant *in vivo* validation simultaneously. This has resulted in a large number of potentially active molecules being stranded in a translational chasm active *in vitro* but uncertain *in vivo* (4–6). This methodological limitation not only restricts the large scale utilization of natural resources but also impedes in-depth mechanistic dissection, leaving natural product sleep research trapped in a cycle of low efficiency trial and error aspects.

To break this bottleneck, there is a pressing need for an efficient research framework that integrates phenotypic profiling, activity screening, and mechanistic validation within a single system. In this review, we leverage the zebrafish as a model organism and, following the systematic logic of “phenotypic profiling → mechanistic mining → component screening → functional validation,” propose a zebrafish -based comprehensive translational platform for evaluating the sleep regulatory effects of natural products. This platform not only addresses the structural gap between high throughput discovery and *in vivo* validation but also provides a replicable, quantifiable new paradigm for plant derived natural products in sleep intervention. Ultimately, this review elevates natural products from isolated chemical activity exploration to systematic functional validation, laying the foundation for future precision based, controllable sleep intervention strategies.

In summary, this article is a narrative review that synthesizes current zebrafish applications in sleep research and natural product evaluation, while proposing a conceptual tiered translational framework that integrates zebrafish with AI-assisted analytics, human organoid validation, and rodent confirmatory studies. It is neither a systematic review nor a detailed methodological protocol; the proposed pipeline is presented as a hypothesis-generating roadmap, as it remains largely untested as an integrated whole. The specific aims are fourfold: (1) to establish the evolutionary and translational basis for using zebrafish in sleep research (Section 2); (2) to evaluate zebrafish-based screening of natural sleep-modulating products and clarify their complementary role alongside rodent models (Section 3); (3) to examine how zebrafish contribute to mechanistic dissection and preclinical prioritization, including relevant methodological standards (Section 4); and (4) to delineate the model’s limitations and outline a staged, next-generation validation platform (Sections 5 and 6).

## 2 Evolutionary conservation of the zebrafish sleep model and its translational basis

As a genetically tractable vertebrate model organism that is highly amenable to genetic manipulation and experimental intervention, the zebrafish has been extensively used in sleep research. Its predictive validity for mammalian responses is rooted in the deep evolutionary conservation of sleep regulatory mechanisms (7, 8). This conservation is evident not only at the level of behavioral phenotypes but also permeates core neural circuits, neurochemical systems, and molecular clock mechanisms, thereby providing a solid foundation for natural product functional screening and pharmacological translation (9–11).

### 2.1 Cross species conservation of sleep behavior and homeostatic regulation

**Figure 1:**
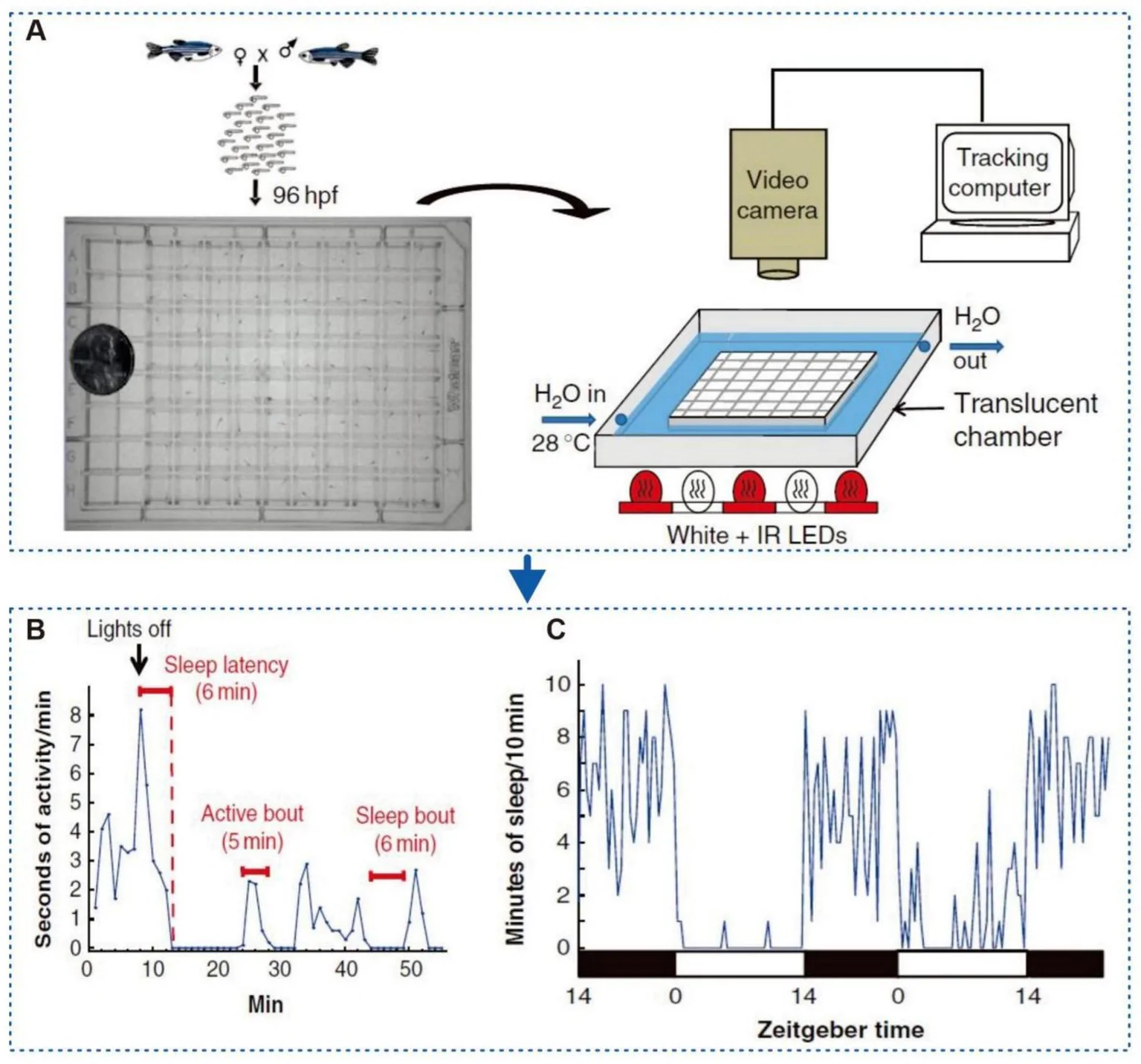
High throughput video tracking platform for quantifying sleep/wake behavior in zebrafish. **(A)** Schematic of the recording setup. Zebrafish larvae older than 96 hpf are placed individually into the wells of a 96-well plate filled with blue water. The plate is housed in a translucent chamber that is continuously illuminated from below by infrared (IR) light emitting diodes (LEDs)—permitting uninterrupted tracking throughout both the light and dark phases—and illuminated by white LEDs from 9:00 a.m. to 11:00 p.m. to impose a 14 h:10 h light dark cycle. Water heated to 28 °C is continuously circulated through the plate chamber to maintain stable temperature and humidity. Behavior is recorded by a video camera connected to a computer running video tracking software; the chamber, lights, and camera are enclosed in a light tight box (not shown) to prevent extraneous light from interfering with the experiment. **(B)** Representative image of an individual larva within a single well, as captured by the camera during tracking. **(C)** Sleep behavior of the same fish shown in panel **(A,B)**, plotted as minutes of sleep per 10-min bin across the light dark cycle; sleep occurs maximally during the night periods, consistent with the diurnal rest activity pattern of zebrafish. In larvae, sleep is operationally defined as bouts of immobility lasting ≥60 s, which are associated with an elevated arousal threshold and homeostatic rebound after deprivation. Reproduced from (17) with permission from Elsevier.

Zebrafish exhibit sleep behavior that is highly congruent with that of mammals, including sustained behavioral quiescence (typically defined as > 6 s of continuous immobility in adult zebrafish or at least 60 s in larvae), an elevated arousal threshold (a markedly reduced response to external stimuli, such as mechanical or electric shock during sleep), and compensatory rebound following sleep deprivation (i.e., sleep homeostasis) (7, 12–17) (Figure 1). Zebrafish are a diurnal species: they are active mainly during the light phase and consolidate their principal rest period during the dark phase, which corresponds to the human nocturnal sleep phase and making them an ideal model for studying the regulation of sleep by photoperiod and circadian rhythms (15, 18). The presence of sleep homeostatic mechanisms in zebrafish indicates that they can maintain equilibrium by modulating sleep pressure and sleep demand, suggesting that fundamental sleep drive mechanisms are highly conserved across vertebrates (19, 20).

### 2.2 Highly conserved neurochemical regulatory networks

Zebrafish retain functional architectures homologous to mammals across key neurochemical systems, including monoaminergic, GABAergic, hypothalamic neuropeptide, and melatonin pathways (13).

Within the monoaminergic system, noradrenergic (locus coeruleus homolog), dopaminergic, and serotonergic (raphe nuclei homolog) neurons have been definitively mapped in zebrafish (21, 22). Pharmacological manipulations that produce arousal or sedation related effects in mammals elicit analogous behavioral outcomes in zebrafish (23, 24). In the GABAergic system, the distribution of GABA_A receptors and of GABAergic neurons is highly conserved; benzodiazepines and non-benzodiazepine GABA_A receptor agonists (the so-called Z-drugs, e.g., zolpidem) reliably induce sleep like behavior in zebrafish, demonstrating cross species conservation of this key pharmacological target (25–28). The hypothalamic orexin/hypocretin system, a core neuropeptide network that regulates wakefulness is functionally intact in zebrafish. Loss of orexin neurons or antagonism of their receptors increases sleep, phenocopying the mammalian response (15, 29, 30).

Regarding the melatonin system, the synthesis, secretion, and receptor signaling pathways are fully functional in zebrafish. Exogenous melatonin robustly enhances nighttime sleep, confirming its conserved role in circadian timing and sleep phase regulation (31–33).

Nevertheless, it is important to acknowledge an inherent limitation. Zebrafish lack a direct homolog of the mammalian neocortex; consequently, they cannot be used to assess how natural products modulate cortical oscillations, i.e., sleep spindles, slow waves or model the prefrontal cortex dysfunction often associated with human insomnia (34–36). This constraint means that candidate sleep regulating functions dependent on cortical circuits must undergo secondary validation in mammalian models.

### 2.3 Cross species conservation of the circadian molecular clock

The zebrafish circadian rhythm is driven by a highly homologous transcription translation feedback loop composed of core clock genes (*clock, bmal, per, cry*), which are expressed in central and peripheral tissues and are sensitive to light dark cycles (37, 38). This molecular level conservation ensures precise regulation of the sleep wake cycle within a 24-h framework, providing a reliable set of molecular targets and functional readouts for studying how natural products may stabilize or reset the biological clock (39, 40).

### 2.4 Genomic conservation and drug-metabolism considerations

Beyond neurotransmitters, sleep patterns, and the circadian cycle, two further dimensions are critical for evaluating the translational validity of the zebrafish model: genomic relatedness and drug metabolism. At the genomic level, analysis of the zebrafish reference genome indicates that approximately 71.4% of human protein coding genes possess at least one zebrafish ortholog, and that roughly 82% of human disease associated genes listed in OMIM have a zebrafish counterpart (41–44). This broad orthology extends to most sleep and circadian relevant loci and underpins the cross-species transferability of genetic and pharmacological findings. Two caveats nonetheless deserve emphasis. First, the teleost specific whole genome duplication means that many mammalian single copy genes are represented by two paralogs in zebrafish, which can subdivide or redistribute ancestral functions and complicate direct genotype phenotype extrapolation (45). Second, differences in gene regulation and isoform usage mean that orthology at the sequence level does not guarantee identical physiological roles; functional validation in the fish is therefore still required for each target of interest.

With respect to drug metabolism and pharmacokinetics, zebrafish possess functional cytochrome P450 monooxygenases, conjugation enzymes, and drug transporters that mediate xenobiotic biotransformation, but the complement of CYP isoforms and their relative activities differ from those of humans, and—because zebrafish are ectothermic—their basal metabolic rate, drug distribution volume, and clearance kinetics are not directly comparable to mammalian values (46, 47). Moreover, the dominant exposure route in larval screens is passive uptake from the surrounding water, which differs fundamentally from the oral or enteral routes relevant to nutritional interventions: compound uptake depends on lipophilicity, molecular weight, and water solubility, and internal exposure concentrations can deviate markedly from nominal bath concentrations. Quantitative pharmacokinetic modeling in zebrafish larvae has nevertheless shown that uptake and clearance can be measured and modeled on an allometric basis, enabling internal exposure estimates that strengthen cross-species extrapolation (48). In practical terms, activity and dose data obtained in zebrafish should be interpreted as qualitative trends and preliminary estimates of effective concentrations rather than as direct equivalents of human effective doses, and definitive dose setting requires pharmacokinetic and pharmacodynamic studies in mammalian models. These genomic and pharmacokinetic considerations are summarized alongside the other conservation dimensions in Table 1.

**Table 1:** Comparative conservation of sleep regulatory mechanisms between zebrafish and mammals.

Regulatory dimension	Zebrafish features	Corresponding mammalian systems	Evidence and significance for conservation	References
Behavior and homeostasis	Behavioral quiescence, elevated arousal threshold, and rebound after sleep deprivation.	Slow wave sleep, rapid eye movement (REM) sleep, and sleep homeostasis	Defines core, cross species features of sleep; supports zebrafish as a model for studying fundamental sleep mechanisms	(7, 15, 16)
Neurotransmitter systems	Intact monoaminergic, GABAergic, orexinergic, and melatoninergic systems	Locus coeruleus, raphe nuclei, basal forebrain, lateral hypothalamus, pineal gland	Conserved responses to classic sedative hypnotics; establishes predictive value for neuropharmacology	(21, 26, 29, 31)
Molecular clock genes	Core clock genes (*clock, bmal, per, cry*) form a feedback loop	Master clock in the suprachiasmatic nucleus (SCN) and peripheral tissues	Provides highly homologous molecular targets for studying natural product modulation of circadian rhythms	(37, 39)
Genome and gene orthology	~71.4% of human protein-coding genes have at least one zebrafish ortholog; ~82% of OMIM disease genes have a zebrafish counterpart	Human reference genome; disease-gene databases (OMIM)	Supports cross-species transferability of genetic and pharmacological targets while flagging paralog-related divergence as a limitation	(41, 43, 44)
Drug metabolism and pharmacokinetics	Functional CYP monooxygenases, conjugation enzymes and transporters	Human CYP isoform complement; oral/enteral absorption; mammalian allometric scaling	Defines quantitative boundaries: zebrafish yield qualitative trends and preliminary concentration estimates; definitive dose setting requires mammalian PK/PD	(46, 47)
Cortical function	Lacks a direct homolog of the neocortex	Prefrontal cortex, thalamocortical circuits	Cannot model cortical oscillations or cognitive aspects of sleep; requires follow up validation in other models.	(35, 36)

**Figure 2:**
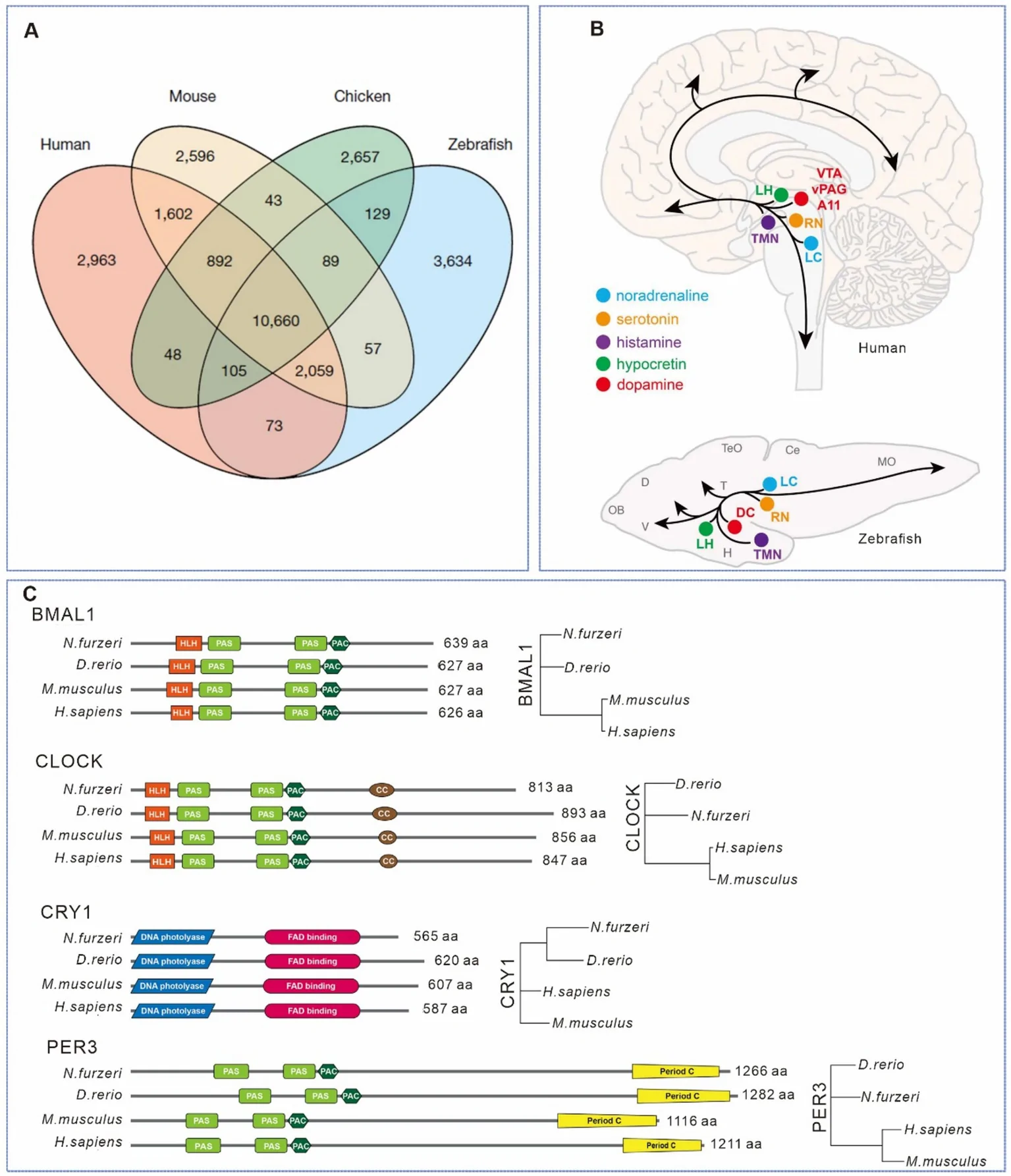
Evolutionary conservation between zebrafish and mammals at the genomic, circuit, and molecular-clock levels. **(A)** Orthologue genes shared between the zebrafish, human, mouse, and chicken genomes. Genes shared across species are considered in terms of copies at the time of the split: for example, a gene that exists in one copy in zebrafish but has been duplicated in the human lineage is counted as only one shared gene in the overlap. The large shared gene core illustrates the broad genomic orthology that supports cross species translation. Reproduced from (28), licensed under CC BY-NC-SA 3.0. **(B)** Neuromodulatory systems that promote arousal in vertebrates. The approximate locations of key neuromodulatory regions are shown for the human and larval zebrafish brains. Arrows indicate ascending projections that increase forebrain excitation and descending projections that increase muscle tone and sensorimotor function. Neuromodulatory regions: LC, locus coeruleus; RN, raphe nuclei; VTA, ventral tegmental area; vPAG, ventral periaqueductal gray; A11, mammalian dopamine cell group A11; DC, dopaminergic diencephalic cluster; TMN, tuberomammillary nucleus; LH, lateral hypothalamus. Larval zebrafish brain anatomy: OB, olfactory bulb; D, dorsal telencephalon; V, ventral telencephalon; TeO, optic tectum; H, hypothalamus; T, thalamus; Ce, cerebellum; MO, medulla oblongata. Note that the human and larval zebrafish brains are not depicted to scale. Adapted from (13), licensed under CC BY 4.0. **(C)** High conservation of core circadian clock components among vertebrates. Left: the functional domains of the core clock proteins in four species—*Nothobranchius furzeri* (turquoise killifish), *Danio rerio* (zebrafish), *Mus musculus* (mouse), and *Homo sapiens* (human)—based on the translated amino acid sequences of BMAL1, CLOCK, CRY1, and PER3. Right: phylogenetic trees constructed using neighbor joining clustering. Reproduced from (38), licensed under CC BY-NC-ND 4.0.

In summary, the zebrafish retains a “universal grammar” of sleep regulation that is highly congruent with mammals across behavioral outputs, neural architecture, and molecular mechanisms, and it exhibits a markedly overlapping response profile to classical sedative hypnotics (49). At the genomic level, zebrafish share a large set of orthologous genes with human, mouse, and chicken (Figure 2A) (28). At the circuit level, the major arousal promoting neuromodulatory systems—the noradrenergic locus coeruleus, serotonergic raphe nuclei, dopaminergic VTA/A11 and PAG, histaminergic tuberomammillary nucleus, and orexinergic lateral hypothalamus—together with their ascending projections that increase forebrain excitation and descending projections that increase muscle tone and sensorimotor function, are mapped onto both the human and larval zebrafish brain (Figure 2B) (13). At the molecular level, a comparison of the functional domains of the core clock proteins BMAL1, CLOCK, CRY1, and PER3 across turquoise killifish, zebrafish, mouse, and human, together with neighbor joining phylogenetic trees for each protein (Figure 2C), reveals conserved domain architecture and short phylogenetic distances, confirming that the molecular machinery of the circadian clock described in Section 2.3 is highly conserved among vertebrates (38).

## 3 Application of the zebrafish model in screening natural products for sleep modulating activity

Leveraging its highly conserved sleep regulatory mechanisms, optical transparency, and rapid developmental cycle, the zebrafish has emerged as a high throughput, cost effective, and translationally relevant model for functional screening of natural products (50–53). Its unique advantage lies not only in the direct observability of behavioral phenotypes but also in its capacity to integrate molecular targets, neural circuits, and circadian rhythms into quantifiable readouts, thereby providing a cutting edge platform for the discovery and pharmacological validation of bioactive compounds from plant and microbial sources (54, 55).

### 3.1 Rapid behavioral phenotyping and quantification

Parameters, such as behavioral immobility, swimming trajectories, activity frequency, and post deprivation sleep rebound enable high throughput screening of natural products for sleep modulatory effects (26, 48). Automated video tracking coupled with software analysis enables simultaneous measurement of sleep duration, arousal threshold, and circadian changes across hundreds to thousands of samples, markedly enhancing screening efficiency (49). Moreover, the diurnal nature of zebrafish aligns their rest phase with human nocturnal sleep, offering an inherent advantage for predicting the efficacy of natural products in improving nighttime sleep (50).

Beyond sleep duration and latency, high density local field potential recordings or dynamic arousal threshold assays in zebrafish can assess the impact of natural products on sleep depth (13, 56). For instance, quantifying the minimal stimulus intensity, such as mechanical or vibrational required to elicit arousal provides a measure of sleep stability (57). This approach more closely mirrors clinical concerns regarding sleep quality and furnishes a direct metric for screening molecules that enhance sleep continuity.

### 3.2 Neurochemical and pharmacological mechanism validation

The zebrafish model enables validation of the neurochemical mechanisms underlying natural product effects through targeted pharmacological antagonists or genetic perturbations. For example, interventions in monoaminergic systems can determine whether a natural product modulates arousal states via dopamine, norepinephrine, or serotonin pathways (58–60). GABA_A receptor antagonism can assess whether its sleep promoting effect depends on this classical inhibitory pathway. Manipulation of the orexin/hypocretin neuropeptide system can reveal potential actions on arousal regulation and circadian rhythms (61). Melatonin pathway experiments can further validate molecular targets associated in circadian resetting or sleep phase shifting (62, 63). This dual behavior, neurochemical validation strategy positions the zebrafish as a complete bridging model for mechanistic pharmacological dissection of natural products, enabling a closed loop analysis from phenotypic observation to molecular mechanisms.

### 3.3 High throughput and multidimensional integrative assessment

The high throughput advantages of zebrafish in natural product screening stem from several complementary features (28, 64–66). Their small size and low maintenance cost enable systematic, large-scale screening in multiwell plate formats, even when natural product resources are limited. Automated behavioral tracking, combining video based monitoring with machine learning, permits rapid, parallel quantification of sleep duration, arousal threshold, and homeostatic rebound following sleep deprivation (67–69), delivering a screening throughput that is difficult to achieve in mammalian models. Beyond behavioral endpoints, the optical transparency and genetic tractability of zebrafish embryos allow optogenetics, calcium imaging, and real-time fluorescence labeling to be coupled directly with behavioral analysis (16, 25, 70–73), enabling visualization, quantification, and causal interrogation of natural product effects on sleep-related neural circuits *in vivo*. In parallel, circadian clock gene reporter systems provide real-time molecular readouts of compound effects on circadian rhythms, facilitating the identification of natural products that can entrain or stabilize the circadian clock (74, 75). By integrating behavioral, circuit-level, and molecular dimensions into a unified workflow, zebrafish support a systematic evaluation of safety, efficacy, and mechanism of action that spans the entire pipeline from primary discovery and target validation to mechanistic dissection (76–78), thereby improving both the predictive accuracy of translation to mammalian models and the reproducibility of sleep pharmacology research on natural products.

### 3.4 Translational value and future prospects of the zebrafish model

The multilevel integration of behavioral, molecular, and pharmacological readouts, the zebrafish model not only enables rapid screening of natural products for sleep modulating activity but also provides a high confidence predictive basis for subsequent mammalian validation and clinical development (79, 80). In the future, combined with AI assisted behavioral analysis, large scale compound library screening, and precision molecular labeling techniques, the zebrafish could serve as a useful translational platform for the discovery, mechanistic dissection, and functional validation of natural sleep modulators (81–83) (Table 2).

**Table 2:** Integrated core advantages of the Zebrafish model for sleep research with natural products.

Core advantage	Technical basis	R&D bottleneck addressed	Key data and outputs	References
High throughput primary screening capacity	Miniaturized larvae, 96 and 384 well plate culture, automated behavioral tracking	Traditional mammalian models cannot handle large scale natural product libraries.	Multidimensional sleep phenotype data enabling rapid ranking and preliminary classification of active compounds	(26, 55)
High content mechanistic dissection capability	Larval transparency, transgenic fluorescent labeling, *in vivo* calcium imaging, optogenetics	*In vitro* models cannot resolve complex neural circuit activity	Dynamic activity maps of sleep/wake related neuronal populations and causal, circuit level evidence	(16, 58, 70)
Early safety warning capability	Easily observable cardiac, morphological, developmental, and locomotor phenotypes	Toxicity is identified only at late developmental stages, incurring high costs.	Simultaneous multi-organ toxicity readouts, enabling parallel early assessment of efficacy and safety	(108–110)
Genetic tractability for target validation	Well established CRISPR gene editing and transgenic line resources	Poorly defined targets of natural products, especially complex extracts	Confirmatory genetic level evidence, elevating mechanistic studies to causal inference	(12, 77, 92)

### 3.5 Comparative rationale: why zebrafish alongside—not instead of—rodent models

The zebrafish is not intended to displace rodent models, and its value does not rest solely on conserved neurotransmitter systems or neural connectivity. Rodents offer undisputed advantages for late-stage confirmation: closer pharmacokinetic similarity to humans (comparable CYP isoform profiles, oral bioavailability, and allometric dose translation), and neuroanatomical fidelity critical for sleep studies (laminated neocortex, thalamocortical oscillations, and EEG signatures directly comparable to human polysomnography) (84, 85). Accordingly, our framework assigns rodent models this confirmatory role (Sections 5 and 6). The rationale for prioritizing zebrafish at the discovery stage, by contrast, derives from a distinct set of capabilities particularly salient for natural product research: (i) Throughput and library scale —zebrafish larvae are compatible with 96 and 384 well formats, enabling *in vivo* screening of hundreds to thousands of samples per week, a scale economically and ethically prohibitive in rodents, yet essential given that natural product libraries are typically large and chemically heterogeneous (86). (ii) Compound economy —larval assays require microgram to milligram quantities, whereas rodent studies demand gram-scale supplies often unavailable for scarce or difficult to purify plant constituents (87). (iii) Optical access —larval transparency permits non-invasive, whole brain imaging of circuit-level drug effects, which is infeasible in rodents without invasive procedures (88, 89). (iv) Genetic tractability and cost —transgenic reporter lines and CRISPR-based validation are faster and cheaper in zebrafish, with per-data-point maintenance costs one to two orders of magnitude lower (90). (v) Ethical and regulatory considerations —larvae younger than 5 days post-fertilization are exempt from many animal welfare regulations, supporting 3Rs-compliant primary screening (91). In short, zebrafish are prioritized where the bottleneck is scale and mechanistic triage; rodents, where the bottleneck is pharmacokinetic fidelity and cortical relevance. The two models thus function as complementary nodes in a single pipeline, not as competitors.

## 4 Zebrafish models for mechanistic dissection and translational potential of natural sleep modulating products

**Figure 3:**
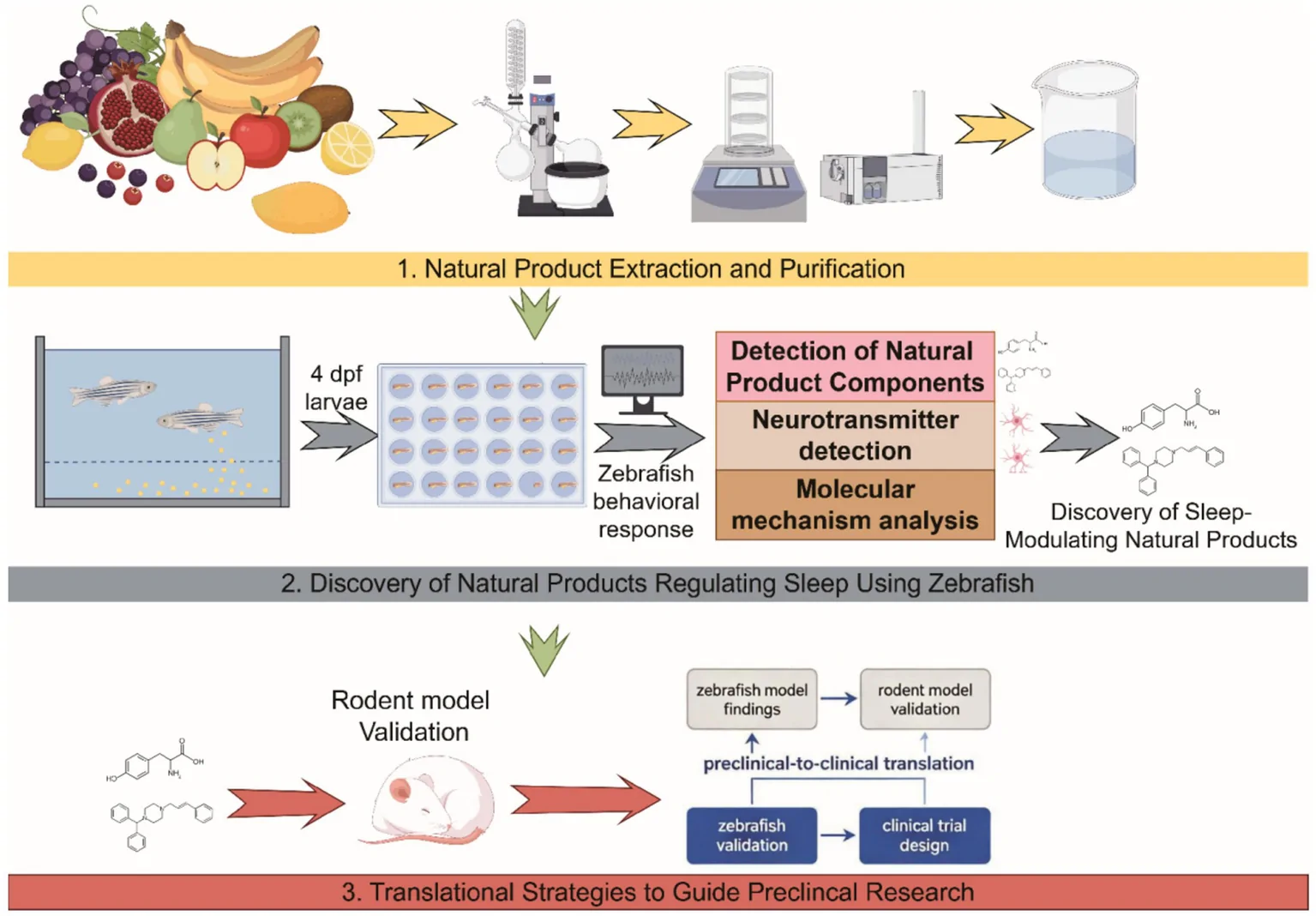
Schematic overview of the workflow for discovering and validating sleep modulating natural products. The pipeline comprises three stages: (1) extraction and purification of natural products from food-derived sources (e.g., fruits and berries) via chromatographic and spectroscopic techniques; (2) high throughput screening in zebrafish (*Danio rerio*) larvae at 4 days post-fertilization (dpf), in which behavioral responses are recorded to identify candidate compounds, followed by neurotransmitter profiling and molecular mechanism analysis to pinpoint sleep modulating natural products; and (3) translational strategies guiding preclinical research, whereby findings from the zebrafish model are validated in rodent models and inform clinical trial design, thereby bridging preclinical to clinical translation. Created by FigDraw.com.

Beyond being an ideal model for studying sleep behavior, the zebrafish offers unique advantages in elucidating the pharmacological mechanisms of natural products and predicting their clinical translatability. The pipeline is schematized as three sequential stages (Figure 3). In the first stage, natural products are extracted and purified from food derived sources (e.g., fruits and berries) using chromatographic and spectroscopic techniques, yielding chemically defined test materials; the library-quality standards required at this stage are detailed in Section 4.3. In the second stage, high throughput screening is performed in zebrafish larvae at 4 dpf: behavioral responses are recorded to identify candidate compounds, and the two analysis streams shown in the figure—neurotransmitter profiling and molecular mechanism analysis—then link the behavioral phenotype to its underlying mechanism, thereby pinpointing sleep modulating natural products and their targets (Sections 4.1 and 4.2). In the third stage, findings from the zebrafish model are validated in rodent models and used to inform clinical trial design, thus bridging preclinical and clinical research (Sections 4.3 and 5.1). The numbered, unidirectional progression across the three stages underscores that advancement through the pipeline is strictly stage gated: a candidate proceeds only after successfully passing the preceding stage. By integrating high throughput screening capacity, quantifiable behavioral phenotypes, and molecular tractability, the zebrafish serves as a bridging model from fundamental discovery to preclinical validation (92, 93). It must be reiterated, however, that all findings derived from zebrafish models constitute testable hypotheses, not direct evidence of equivalent therapeutic efficacy in mammals.

### 4.1 The complete mechanistic dissection of pharmacological action

The zebrafish model permits multilevel, simultaneous analysis of natural product mechanisms at the molecular, cellular, and behavioral scales (94–96). With respect to neurotransmitter and receptor targets, neurochemical profiling —here meaning the quantitative measurement of neurotransmitters and their metabolites (e.g., GABA, serotonin, dopamine, and their turnover products) in zebrafish brain tissue by HPLC or LC–MS/MS, combined with receptor-level pharmacological blockade— can precisely localize the modulatory effects of natural products on core sleep regulating targets, i.e., GABA_A, serotonin, melatonin, and orexin/hypocretin (97–99). At the level of hypothalamic and brainstem neural circuits, combining *in vivo* calcium imaging and optogenetics —i.e., transgenic expression of light-gated ion channels (such as channelrhodopsin-2) or pumps in defined neuronal populations so that these neurons can be activated or silenced with millisecond precision by light— permits real time observation of natural product induced alterations in sleep wake circuit activity, providing dynamic and quantitative evidence for mechanistic dissection (8, 58, 100–102). In the way of gene expression and signaling pathways, the use of transgenic zebrafish (lines carrying fluorescent reporters under pathway specific promoters, which make signaling activity visible *in vivo*) and CRISPR technology (targeted gene knockout or knock in to test whether a candidate molecular target is necessary for the observed effect) enables exploration of key molecular pathways, such as NFκB, JAK/STAT, and cAMP/PKA through which natural products regulate sleep, and further reveals their potential neuroimmune endocrine integrative effects (103, 104). This multi-layer dissection strategy overcomes the high cost and low throughput limitations of traditional mammalian models, while generating mechanistic data that can directly guide subsequent validation in rodent models.

### 4.2 Translational strategies to guide preclinical research

Pharmacological mechanism data obtained from zebrafish can be used to build predictive models associated to natural products, their targets, and behavioral phenotypes, thereby supporting preclinical research (105–107). For dose prediction and safety assessment, zebrafish enable rapid evaluation of safe dose ranges and potential toxicity, providing an initial dose reference for mammalian studies; however, the definitive therapeutic dose must be determined through rodent pharmacokinetic and pharmacodynamic investigations (108–110). For validating multitarget interactions, natural products often modulate sleep through pleiotropic mechanisms, and the zebrafish model can dissect their integrated regulatory effects on neurotransmitters, neuropeptides, and immune factors (111, 112). For hypothesis generation, the combination of *in vivo* imaging and genetic tools provides unambiguous evidence of mechanistic pathways, generating testable hypotheses for subsequent validation in mammalian models (113, 114). For accelerating lead compound discovery, high throughput zebrafish screening can rapidly identify natural product candidates with genuine clinical development potential (115, 116) (Table 3).

**Table 3:** Integrated zebrafish technology roadmap for dissecting the sleep modulating activity of natural products.

Research layer	Core technical methods	Key readout parameters	Objectives and outputs	References
Behavioral phenomics screening	Automated video tracking, long term continuous monitoring, and arousal threshold determination	Total sleep time, sleep onset latency, sleep fragmentation index, sleep depth, circadian rhythm parameters	Establish behavioral fingerprints of compounds; enable high throughput activity screening and preliminary mode of action classification.	(57, 67, 75)
Neural activity and circuit interrogation	*In vivo* calcium imaging, fiber photometry, optogenetics, chemogenetic manipulation	Dynamic calcium signals in defined neuronal populations; behavioral reversal under optogenetic control	Visualize real time compound effects on neural circuits; validate the necessity of specific circuits for observed behavioral effects.	(16, 71, 100)
Molecular targets and signaling pathways	Selective receptor antagonists/agonists, CRISPR gene editing, transcriptomics, and proteomics	Pharmacological blockade effects; activity changes in knockout lines; differentially expressed genes and pathway enrichment	Identify direct molecular targets; generate hypotheses for novel signaling pathway mechanisms.	(26, 97, 104)

### 4.3 Practical applications of the zebrafish model in natural product sleep research

The study using a zebrafish larval insomnia model demonstrated that *Centella asiatica* extract effectively improves sleep behavior (98). Using a randomized, controlled post test design, the authors divided zebrafish larvae into a normal control group, an insomnia model group, and groups treated with different concentrations of *Centella asiatica* extract (2.5–10 μg/mL). The locomotor activity of zebrafish larvae was recorded using Basler cameras at 5, 6, and 7 days post-fertilization (dpf), and subsequent western blot analysis revealed that the extract significantly suppressed the expression of orexin and p38 proteins and reduced the phosphorylation levels of ERK and Akt. The 5 μg/mL concentration showed the strongest inhibition of orexin and p38, while 2.5 μg/mL was most effective in reducing ERK and Akt phosphorylation. Behavioral results indicated that the *Centella asiatica* extract prolonged total immobility time and shortened the first sleep latency, suggesting sleep improvement via modulation of orexin and related signaling pathways. This study highlights the utility of the zebrafish model for dissecting the neuroregulatory mechanisms of natural products.

An extract of *Ziziphi Spinosae* semen effectively restored daytime locomotor activity in sleep deprived zebrafish and significantly elevated levels of GABA, hypocretin, and neuromedin U (117). The sleep modulatory mechanism of the active constituents, jujuboside and jujubogenin, may be attributed to their effects on GABAergic, hypocretinergic, and neuromedin U systems, including influences on GABA receptor subtypes and related gene expression. This research holds significant implications for insomnia intervention and recovery promotion.

Under conditions of sleep disruption induced by nocturnal light exposure, *Aquilaria sinensis* leaf tea (1.0 and 1.5 g/L) significantly upregulated total sleep time in larval zebrafish (78). Transcriptome sequencing and qRT-PCR analysis showed that the expression of immune related genes, including *nfkbiab*, *tnfrsf1a*, *nfkbiaa*, *il1b*, *traf3*, and *cd40*, was downregulated in the treatment groups, while the expression of the sleep associated gene *bhlhe41* was also markedly downregulated. In *Tg(mpo: GFP)* transgenic zebrafish, the leaf tea suppressed light induced neutrophil accumulation, indicating an immunomodulatory effect. These results suggest that the *Aquilaria sinensis* leaf tea ameliorates nocturnal light induced sleep disturbance by modulating immune related pathways, providing empirical support for the application of the zebrafish model in functional screening and mechanistic analysis of natural products for sleep regulation.

In summary, the value of the zebrafish in natural product sleep research can be encapsulated in different key points. Conservation sleep related behaviors, neural circuits, and molecular pathways are highly conserved across mammals; operability the model integrates high throughput screening, behavioral tracking, and molecular dissection; and translational guidance acting as a translational filter, it provides lead prioritization and mechanistic hypotheses for subsequent mammalian models, substantially improving the efficiency and success rate of natural product development (Table 4).

**Table 4:** Representative natural sleep active ingredients and their mechanisms of action identified using the zebrafish model.

Mechanisms	Source	Key evidence in the zebrafish model	Putative or validated underlying pathway	Translational stage	References
Direct neuromodulation	Jujuboside & jujubogenin (from *Ziziphi Spinosae* semen); material tested: enriched extract standardized for jujubosides, with mechanistic attribution to these constituents remaining putative until confirmed with the isolated compounds	Restores daytime locomotor activity in sleep deprived zebrafish; significantly elevates GABA, hypocretin, and neuromedin U levels	Modulates GABAergic, HCRTergic, and NMUergic systems; affects GABA receptor subunit composition and related gene expression	Undergoing rodent validation	(51, 96)
Indirect systemic modulation	*Aquilaria sinensis* leaf tea (from *Aquilaria sinensis* leaves); material tested: crude *Aquilaria sinensis* leaf infusion (a complex mixture), with quercetin and eupalitin proposed as candidate but not yet confirmed active compounds	Increases sleep duration in larval zebrafish under nighttime light induced sleep disruption; downregulates immune related genes (*nfkbiab*, *il1b*, *cd40*); reduces neutrophil infiltration	Improves sleep via immune related pathways (e.g., NF-κB); quercetin and eupalitin are candidate active compounds	Zebrafish study completed; further translation required	(78)
Multitarget synergy	*Centella asiatica* ethanol extract; material tested: crude ethanol extract, with active molecule(s) not isolated and observed polypharmacology possibly reflecting synergistic or additive actions of multiple constituents	Prolongs total immobility time; shortens first sleep latency; suppresses orexin and p38 protein expression; reduces ERK and Akt phosphorylation levels	Modulates orexin and MAPK signaling pathways (ERK, Akt, p38)	Zebrafish study completed; mechanistic dissection ongoing	(98)

### 4.4 Leveraging the zebrafish platform to address pleiotropy and define the natural product library

A recurring challenge in this field is that natural products rarely act through a single target: crude extracts are, in effect, naturally occurring polypharmacological mixtures whose sleep modulating activity emerges from synergistic and additive interactions among constituents. The zebrafish is unusually well suited to dissecting such pleiotropy, for reasons grounded in a mature methodological literature rather than in speculation. First, by approximately 5 days post-fertilization, larvae display robust, quantifiable sleep/wake behavior while remaining optically transparent, permitting whole brain calcium imaging at cellular resolution during unrestrained behavior (16, 83); this behavioral neural coupling allows a compound induced sleep phenotype to be linked directly to the circuit that produces it. Second, large-scale behavioral profiling has shown that neuroactive compounds, including multitarget molecules whose phenotype no single target explains, generate reproducible behavioral “fingerprints” that cluster by mechanism class, enabling mechanism prediction from the *in vivo* phenotype alone (55, 67, 105, 106, 111). Third, a mature genetic toolkit provides a ready made reporter scaffold: well characterized sleep mutants, such as hypocretin/orexin receptor mutants with short and fragmented sleep (15), and genetic screens that identified neuromedin U as a sleep/wake regulator (75), offer defined genetic backgrounds onto which natural product libraries can be superimposed, so that phenotypic rescue or modification rapidly nominates candidate molecular targets. Finally, high throughput zebrafish drug screening simultaneously captures systemic phenotypes and toxicity (26, 109), allowing efficacy and early safety to be read out in parallel. Together, these features extend a proven discovery logic, from single compounds and genes, to the chemically complex mixtures that dominate nutritional intervention research; and, particularly when combined with bioassay guided fractionation (80, 107), they convert the pleiotropy of natural products from an interpretive obstacle into an experimentally tractable variable.

The proposed framework also presupposes a clearly defined, quality controlled natural product library, which we specify here. The library is envisioned as a curated collection of plant and microorganism derived materials, prioritized toward edible species and medicine food homology resources relevant to nutritional applications. Each entry should carry a minimum metadata set: authenticated source material (species, plant part, geographic origin, and voucher information); extraction and fractionation details (solvent system, yield, and batch identifier); and a standardized chemical fingerprint, such as HPLC/UPLC profiling with LC–MS annotation of major constituents, so that every tested sample is chemically traceable rather than an anonymous “extract.” Entries would span a deliberate complexity gradient, from crude extracts through enriched fractions to isolated compounds, allowing mixture level activity to be progressively deconvoluted within the same screening system. These specifications are prerequisites for the “mechanism traceable” and “precision” nutritional intervention claims made in this review: AI based prediction models (Section 5.2) can be trained and audited only if each library entry is linked to structured chemical and provenance data, and cross-species translation is meaningful only when the material tested in zebrafish is the same chemically defined material subsequently advanced to further validation.

### 4.5 Methodological considerations: dose, exposure route, and extract complexity in natural product studies

Natural product studies entail methodological challenges distinct from those encountered with single synthetic compounds, and the examples discussed above are no exception. We therefore highlight three issues that warrant systematic reporting and careful interpretation in zebrafish based natural product sleep research.

First, dose and concentration characterization: nominal bath concentrations (e.g., 2.5–10 μg/mL for *Centella asiatica* extract; 1.0–1.5 g/L for *Aquilaria* leaf tea) do not equate to internal exposure, as compound uptake from the medium depends on lipophilicity and molecular size (78, 98, 118). Where feasible, internal exposure should be analytically verified, and full concentration–response relationships—including the maximally effective and no-effect concentrations—should be reported rather than single-dose observations (119, 120). Second, route of administration: passive waterborne immersion, the predominant exposure route in larval screens, bypasses gastrointestinal digestion, first-pass metabolism, and microbiota-mediated biotransformation—processes that orally consumed functional foods would normally undergo (121–123). When the intended application is nutritional, microinjection, microgavage, or dietary incorporation may constitute more physiologically relevant routes (123); the chosen route should therefore be explicitly stated and justified. Third, extract complexity and potential synergism: as documented in Table 4, several of the reported effects were obtained with crude or enriched extracts rather than isolated compounds. Natural extracts are complex mixtures whose bioactivity may arise from synergistic or additive interactions among multiple constituents, rather than from a single active molecule (124). Attribution of an extract’s effect to a specific constituent (e.g., quercetin or eupalitin in *Aquilaria* leaf tea) should accordingly be regarded as provisional until verified with the isolated compound, tested both alone and in recombined form (78, 124).

Collectively, rigorous attention to these methodological variables is critical for ensuring the reproducibility and translational credibility of zebrafish-based natural product sleep research.

## 5 Limitations, challenges, and future directions: toward a next generation integrated validation platform

### 5.1 Model inherent limitations and a staged translational validation strategy

The translational validity of the zebrafish model rests on a clear understanding of its appropriate application boundaries. Its limitations are not deficiencies but rather define its optimal position within the drug discovery pipeline as a filter (125–127).

Physiological and pharmacokinetic difference: as an ectothermic vertebrate, zebrafish exhibit metabolic rates, drug distribution, and clearance profiles that differ from mammals. Therefore, activity and dose data obtained from zebrafish should be interpreted as qualitative trends and preliminary estimates of effective concentrations, not as direct equivalents to therapeutic doses in mammals (48, 128, 129). Calibration and translation require subsequent pharmacokinetic and pharmacodynamic studies in mouse, rat, or other mammalian models.

Neurological simplicity: the zebrafish brain is relatively simple, notably lacking a structure homologous to the mammalian neocortex. Consequently, it cannot model complex sleep disorders linked to higher order cortical functions in humans. Research in zebrafish should focus on conserved core sleep wake circuits and circadian regulatory mechanisms (130, 131). For insomnia models involving comorbidities, such as depression or anxiety, candidate molecules identified in zebrafish must undergo secondary validation and efficacy confirmation in transgenic or stress induced rodent models that recapitulate these complex pathologies (96, 132, 133).

Immature blood–brain barrier in larva: before 7 days post fertilization, the larval blood–brain barrier (BBB) is not fully developed, potentially leading to overestimation of CNS accessibility for certain macromolecular compounds. Mechanistic studies should therefore assess BBB integrity using Evans blue dye exclusion or similar permeability assays (134, 135). For screens specifically targeting direct CNS action, parallel pre-screening using *in vitro* BBB models, or re-evaluation of primary positive hits in adult zebrafish (with an intact BBB) is advisable.

Limited dimensionality of sleep phenotypes: most zebrafish sleep studies rely primarily on behavioral quiescence, making it difficult to directly assess sleep depth, slow wave activity intensity, or other qualitative features of sleep. This limitation can be addressed by incorporating high density local field potential recordings and dynamic arousal threshold measurements to enrich the phenotypic repertoire (15, 16, 136–138).

However, translation of discoveries made in zebrafish must adhere to a staged validation principle. Zebrafish serve as a powerful primary screen and hypothesis generator; their output must be subjected to rigorous sequential testing, including complex phenotype and efficacy validation in rodent disease models, dose calibration through pharmacokinetic studies, and comprehensive toxicological evaluation to assemble a complete preclinical evidence chain.

### 5.2 Future paradigm: building an intelligent, humanized predictive platform

To transcend current limitations, we envision a future paradigm that integrates cutting edge technologies, with the core aim of elevating the zebrafish from an experimental tool to a key node in the data driven discovery engine.

AI driven deep phenotyping and predictive modeling: future applications of deep learning for unsupervised analysis of high throughput behavioral videos (139–142) promise to uncover subtle, dynamic behavioral patterns beyond traditional pre-defined parameters, such as total sleep time, thereby enabling the generation of unique digital biomarkers. These multidimensional behavioral phenomics data, when combined with compound structural information, can be used to train machine learning models to predict the sleep activity and potential mechanism of action class of novel compounds. This will enable a paradigm shift from empirical screening to closed loop of intelligent prediction and experimental validation.

Cross species data bridging and humanized organoid validation: a key priority is establishing a comparative database that aligns gene expression and pathway activities across zebrafish, mammals, and humans. More importantly, active compounds identified in zebrafish should be tested in human induced pluripotent stem cell (iPSC) derived brain organoids or microfluidic gut–brain axis chips (143–145). Such humanized systems can directly assess compound specificity, potency, and potential toxicity on human neural cells, inserting a critical pre-clinical checkpoint into the translational pipeline and greatly enhancing predictive value.

**Figure 4:**
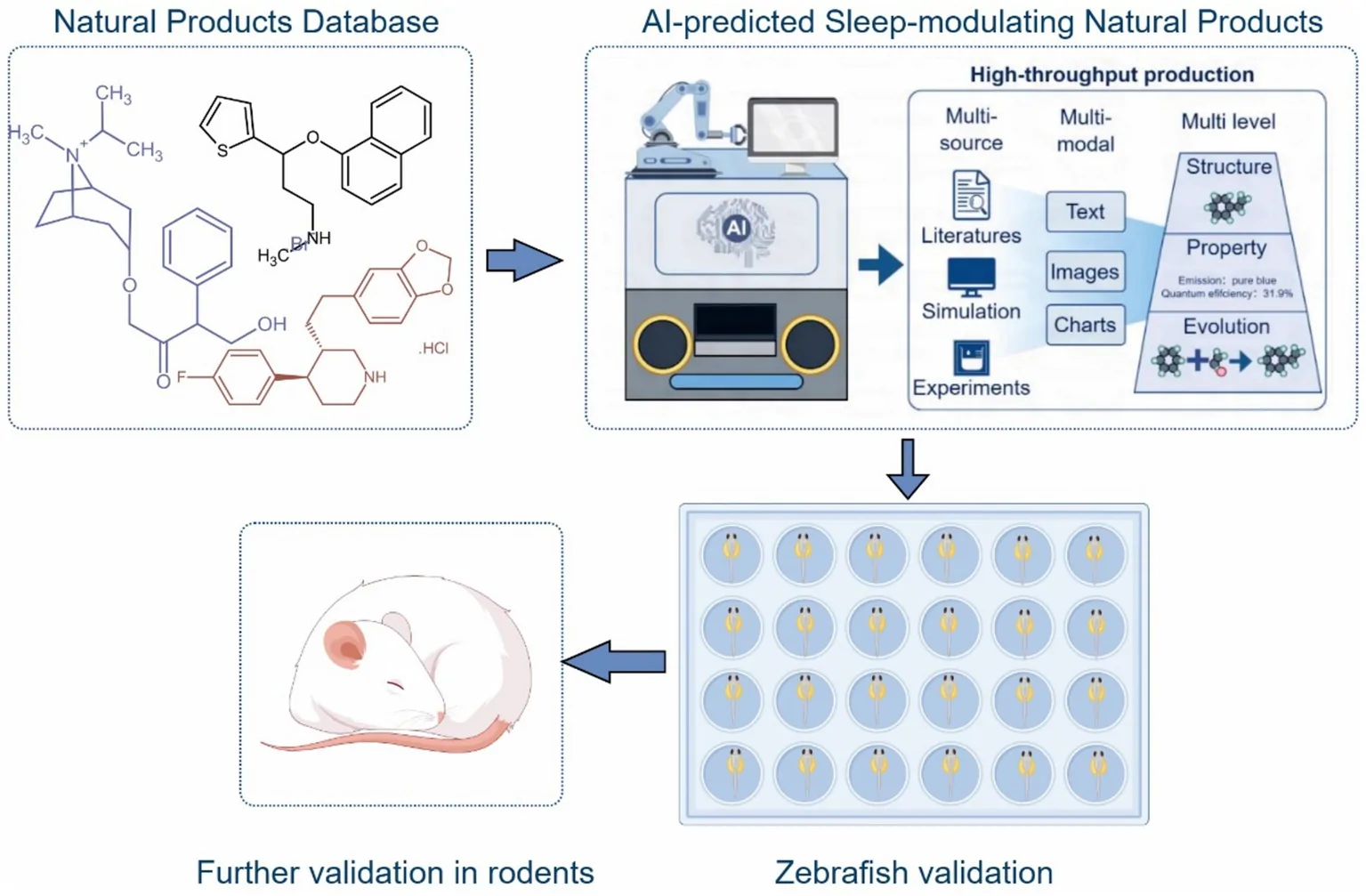
Schematic representation of the artificial intelligence (AI)-driven pipeline for identifying and validating sleep modulating natural products. Candidate compounds retrieved from a natural products database are subjected to high-throughput AI-based prediction, which integrates multi-source data (literature, simulations, and experiments), multi-modal information (text, images, and charts), and multi-level analyses spanning molecular structure, physicochemical and pharmacological properties, and evolutionary relationships. The resulting AI-predicted sleep modulating natural products are first validated using a high-throughput zebrafish screening platform, and the most promising candidates subsequently undergo further validation in rodent models to confirm their sleep regulatory efficacy. For clarity, the schematic depicts the currently established pipeline stages; human organoid/organ-on-a-chip validation (Sections 5.2 and 6) is discussed in the text as an emerging future direction. Created by FigDraw.com.

Open access databases and collaborative ecosystems: the construction of a unified, publicly accessible database for zebrafish sleep phenotypes and molecular responses standardizing storage of compound information, multidimensional behavioral data, imaging, and omics datasets are essential. Open sharing of such high quality datasets will facilitate global data mining, the construction of virtual screening models, and collaborative research, thereby accelerating the accumulation and iteration of knowledge in the field (9, 143, 146–148) (Figure 4). As depicted in Figure 4, these threads converge into a concrete workflow: candidate compounds are first retrieved from a natural products database and subjected to high throughput AI based prediction that integrates multi-source data (literature, simulations, and experiments), multi-modal information (text, images, and charts), and multi-level analyses spanning molecular structure, physicochemical and pharmacological properties, and evolutionary relationships; the resulting AI predicted sleep modulating natural products are then validated using the high throughput zebrafish screening platform described in Sections 3 and 4, and only the most promising candidates proceed to confirmatory validation in rodent models, as laid out in the staged roadmap of Section 6.

## 6 Conclusions and forward look

In the present article, we propose a forward looking, standardized roadmap for the intelligent screening and validation of natural sleep modulators. This roadmap is structured as a tiered, sequential pipeline.

First, zebrafish based high throughput phenomic smart screening serves as the initial entry point. Large scale screening of natural product libraries in zebrafish generates multidimensional behavioral fingerprints, including sleep duration, onset latency, fragmentation index, and arousal threshold. When integrated with AI driven analytics, this approach enables rapid identification of active candidates. It provides an early prediction of their likely mechanism of action, distinguishing, for example, direct neural modulation from indirect systemic regulation.

Second, in-depth mechanistic dissection in zebrafish is performed on positively screened compounds. Leveraging the optical transparency and genetic tractability of zebrafish, this stage employs *in vivo* neural circuit interrogation via calcium imaging, optogenetics, and CRISPR based validation to pinpoint direct molecular targets and signaling pathways. Early toxicity assessment is conducted in parallel, enabling simultaneous profiling of efficacy and safety.

Third, human organoid and organ on a chip validation provides a critical translational checkpoint for human relevance. Mechanistically defined candidates are tested in iPSC derived human brain organoids or multi cell type gut–brain axis chips to evaluate their activity, target specificity, and safety profile within a human cellular context.

Fourth, confirmatory validation in rodent disease models represents the final preclinical stage. Candidates that successfully pass the preceding layers are comprehensively assessed in transgenic or behaviorally induced rodent models that recapitulate key pathological features of specific sleep disorders. These studies evaluate efficacy, pharmacokinetic properties, and long term safety, thereby completing the full preclinical evidence chain.

Collectively, this roadmap delineates a clear, tiered path of progressive enrichment and validation from vast chemical libraries to well characterized preclinical candidates. Zebrafish not only serve as an efficient starting point for this pipeline but also generate standardized, computable data that fuel the training and optimization of the entire intelligent discovery system. By embracing artificial intelligence, humanized model systems, and systems biology, the zebrafish platform is evolving from a powerful model organism into a core experimental and computational engine for next generation natural product discovery in neuropsychiatric indications. If validated in practice, this transformation could help move the discovery of natural sleep enhancing agents toward a precision science defined by data driven workflows, iterative cross modal validation, and transparent translational trajectories.
